# Flavonoids Are Intra- and Inter-Kingdom Modulator Signals

**DOI:** 10.3390/microorganisms10122479

**Published:** 2022-12-15

**Authors:** Elisa Ghitti, Eleonora Rolli, Elena Crotti, Sara Borin

**Affiliations:** Department of Food, Environmental and Nutritional Sciences, University of Milan, Via Celoria 2, 20133 Milan, Italy

**Keywords:** phytochemicals, root exudates, phytobiome, plant-microbe interactions, beneficial microbes, plant secondary metabolites, abiotic stress, biotic stress, rhizosphere, microbiome

## Abstract

Flavonoids are a broad class of secondary metabolites with multifaceted functionalities for plant homeostasis and are involved in facing both biotic and abiotic stresses to sustain plant growth and health. Furthermore, they were discovered as mediators of plant networking with the surrounding environment, showing a surprising ability to perform as signaling compounds for a multitrophic inter-kingdom level of communication that influences the plant host at the phytobiome scale. Flavonoids orchestrate plant-neighboring plant allelopathic interactions, recruit beneficial bacteria and mycorrhizal fungi, counteract pathogen outbreak, influence soil microbiome and affect plant physiology to improve its resilience to fluctuating environmental conditions. This review focuses on the diversified spectrum of flavonoid functions in plants under a variety of stresses in the modulation of plant morphogenesis in response to environmental clues, as well as their role as inter-kingdom signaling molecules with micro- and macroorganisms. Regarding the latter, the review addresses flavonoids as key phytochemicals in the human diet, considering their abundance in fruits and edible plants. Recent evidence highlights their role as nutraceuticals, probiotics and as promising new drugs for the treatment of several pathologies.

## 1. Flavonoids: Plant Secondary Metabolites with a Broad and Diversified Spectrum of Activities across All Life Kingdoms

A diverse set of environmental cues triggers plant production of specialized metabolites in a highly regulated spatial and temporal dynamic [1] aiming to face unfavorable growth conditions [2]. Such phytochemicals are key players in plant defense, protection, adaptation to abiotic and biotic stress and communication with the other members of the phytobiome [3].

Flavonoids form one of the broadest groups of specialized metabolites and include over 9000 derivatives [4]. Beyond the canonical C_6_-C_3_-C_6_ core structure of polyphenolics, the flavonoid family encompasses *in sensu lato* chalcones, flavones, flavonols, anthocyanins and proanthocyanidins [5]. This multiplicity in chemical structure is mainly due to secondary modifications by glycosylation, prenylation, methylation and acylation that affect the bioactive role, transport and accumulation of the resulting compounds [6], a complex set of molecules that enables plants to interact dynamically with the environment. The wide distribution of biosynthetic genes in the plant kingdom, already observed in microalgae, suggests that plants were equipped early during evolution with the ability to produce flavonoids [7]. This ancestral origin may be related to flavonoids’ protective role against biotic threats such as pathogenic fungi and bacteria and herbivore predation and abiotic ones such as ultraviolet (UV) light [8]. It has been hypothesized that the evolutionary success of embryophytes in the Paleozoic as land-colonizers was achieved by the antioxidant activity of phenolic compounds, able to screen for UV radiation [9].

So far, flavonoids are documented to exert a wide spectrum of biological activities in plant physiology [10] and, being differentially produced and regulated in different tissues, organs and cell-types, contribute to a plastic response to environmental stimuli [2]. These phytochemicals have a role even in plant reproduction strategies: they encompass pigmented molecules, such as anthocyanins, that show from blue to red colors, and accumulate in flowers and fruits to attract pollinators and animal dispersers to favor the dispersal of seed [11].

Flavonoids are involved in many plant developmental processes [12,13] and, more precisely, they contribute to stress-induced morphogenic responses under a variety of abiotic conditions [14] including salinity [15], drought [16], heat [17], freezing [18], metal toxicity [19], presence of xenobiotics [20] and UV exposure [21].

Flavonoid aglycones and glycosides are secreted in root exudates [22], acting as communication signals that rule, for instance, plant-to-plant interactions in allelopathic interference between different species [23] and that mediate root association with below-ground soil microbiomes [24,25]. In the soil microbiome framework, the role played by flavonoids in the legume-rhizobia symbiosis has been extensively investigated [26,27,28]. In response to nitrogen deficiency, legumes release species-specific flavonoids as chemical impulses to attract nitrogen-fixing rhizobia and to activate the transcription of bacterial genes coding for Nod factors [29,30]. This, in turn, affects the plant root morphogenetic development, culminating in root infections and nodule organogenesis [31]. Besides bacteria, flavonoids are signaling compounds also for arbuscular mycorrhizal fungi, influencing spore germination, hyphal growth and root colonization efficiency [32], in turn contributing to the plant’s nutritional balance for micronutrients such as phosphorus and iron [33]. Evidence is accumulating that flavonoids could act as a more universal signaling system for soil microbes [34] as suggested by the huge proportion they represent in root chemistry: in *Arabidopsis*, for instance, they constitute 37% of the total secondary metabolites released into the rhizosphere [35]. Flavonoids can be metabolized by microorganisms as nutrients [36], can affect microbial traits involved in rhizocompetence such as motility and chemotaxis [37,38] or operate as co-metabolites to induce highly specialized pathways in bacteria, such as the expression of the *bph* operon involved in the bioremediation of recalcitrant xenobiotics like polychlorinated biphenyls [39,40].

Flavonoids are essential components of food and edible plants and, once introduced through the diet, they are transformed by the human microbiome in the intestine [41]. Parental and digested flavonoids exert their action locally, by sustaining the intestine functionality in terms of detoxification from toxins and inhibition of pathogens, increasing nutrients up-take and maintaining the intestinal barrier integrity [42]. Part of the flavonoid molecules are released in the blood flow where they act systemically. Numerous medical studies highlighted flavonoid therapeutic potential in the treatment of diverse pathologies, paving the way for the development of new drugs or combinatory treatments for human health [43,44].

This review aims to shed light on (i) the multifaceted roles played by flavonoids in extremely diverse biological systems, (ii) their ability, as signal molecules, to facilitate communications among organisms belonging to different life kingdoms, (iii) their plant-beneficial effect in improving resilience to adverse environmental conditions and (iv) their cross-reaction with animal signaling pathways, thus exerting therapeutic effect for the medical treatment of several human acute illnesses.

The source analyses for this review encompassed the scientific literature deposited in online databases such as the Web of Science Core Collection, PubMed, Scopus and Google Scholar, using keywords that describe flavonoids and their involvement in biotic and abiotic stresses (including oxidative stresses, nutrient scarcity, soil pollution and attack by phytopathogens), allelopathy, microbiome assembly and function, root colonization, chemotaxis, biofilm formation, rhizoremediation, insect, oviposition, feeding, quercetin, naringenin, nutraceuticals, dietary phytochemicals, human health and disease. The data critically discussed in the present review were retrieved from studies published in the years from 2002 to 2022 (accounting for 98% of the cited literature).

## 2. Flavonoids Tune Plant Physiology Response under Biotic and Abiotic Stress Conditions

**Figure 1:**
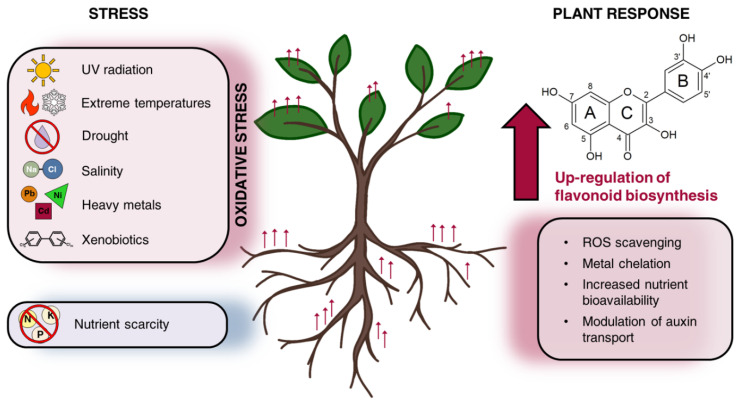
Flavonoid role in plant physiology under abiotic stress. To cope with poor nutrient availability and a variety of abiotic stresses featured by oxidative stress, plants trigger an enhanced synthesis of flavonoids that contributes to tune plant homeostasis and development. A schematic representation of the main structural components and substituent positions in the flavonoid backbone is reported in the image.

Due to their structural heterogeneity, flavonoids play multiple roles in mediating plant physiology and in responding to different abiotic stresses (Figure 1).

The same flavonoid can have multiple functions and be involved in plant responses to high salinity [45], drought stress [46], UV radiation [47], extreme temperatures [48], the presence of heavy metals in soils [49] and nutrient scarcity [10,50]. Therefore, despite the energetically highly-consuming process of their synthesis, flavonoids’ versatility contributes to plant terrestrialization [51] and represents an advantageous molecular resource to maintain plant homeostasis, especially under stress conditions [52,53]. Acting as reactive oxygen species (ROS) scavengers is one of the main functions of these molecules, an essential feature to mitigate the damages caused by environmental conditions that trigger oxidative stress [54,55,56]. ROS-scavenging flavonoids are an essential defense to counteract oxidative damage, such as DNA and protein oxidation and lipid peroxidation [57], supporting the activity of plant antioxidant enzymes [58,59]. The antioxidant activity of flavonoids is related to their polyphenolic structure and to the presence of a wide variety of attached substituents, such as catechol groups on the B ring and hydroxyl groups [60] (Figure 1). For these reasons, flavonoids such as quercetin and myricetin showed a stronger antioxidant capacity than kaempferol [61]. This type of structure allows the efficient quenching of free radicals and singlet oxygen, the chelation of metal ions such as Fe^2+/3+^ and Cu^2+^, essential to prevent ROS generation [62] and the repression of the activity of ROS-producing enzymes such as oxygenases and oxidases [63]. Quercetin derivatives, in particular, are involved in detoxification under different oxidative stresses caused by intense light and drought, because they possess the ideal structural requirements as dihydroxy-B-ring-substituted compounds. Moreover, their biosynthesis is crucial for plant fitness [64].

In the context of food quality, growth in a condition of oxidative stress is sometimes sought in edible plants to increase their content in beneficial antioxidant metabolites such as flavonoids: the leafy vegetable *Amaranthus tricolor*, naturally endowed with antioxidant pigments and metabolites, enhanced its total flavonoid content and antioxidant capacity when subjected to salinity stress by irrigation with 50–100 mM NaCl water solution [45]. Similarly, the application of short-term high light irradiation with LED lights on hydroponic lettuce improved its nutritional quality and induced a higher accumulation of antioxidant flavonoids and anthocyanins [65].

Exposure to excessive UV radiation is indeed one of the main triggering conditions for oxidative stress in plants, leading to DNA breaks, cell damage and impairment of the photosynthetic process [14,50]. To counteract UV stress, increased biosynthesis of specific types of flavonoids is induced to limit excessive light radiation damage, acting in synergy with antioxidant enzymes, the plant main defense strategy against ROS [66]. This is the case of UV light-induced quercetin 3-*O*- and luteolin 7-*O*-glycosides that were observed to act as O_2_^−^ scavengers in *Ligustrum vulgare* exposed to UV-B radiation [67]. Also, apigenin biosynthesis was enhanced under UV-B stress in transgenic *Arabidopsis* plants expressing maize flavone synthases, leading to reduced membrane damage explained by the role of flavonoids in the reduction of lipid peroxidation [68]. Besides showing higher accumulation of flavonoids, stressed plants exhibit a marked up-regulation of genes involved in their biosynthesis: in UV-B-exposed hairy roots of *Isatis tinctoria* L. the level of gene transcription for chalcone synthase was 405-fold higher than the control, together with a 16.5-fold increase in flavonoid levels [69]. In the mutant line *tt4* of *Arabidopsis thaliana*, the knock-out of the chalcone synthase gene leads to the inability to synthesize kaempferol, therefore making the plant more sensitive to UV-light exposure [14].

Regarding their location in plant tissues, light-induced flavonoids such as flavonols and anthocyanins are not present exclusively in leaf epidermal cells or external appendages such as trichomes, where they can prevent ROS formation through a photoprotective action as UV-shelter molecules [68,70]. Since their beneficial function is mostly related to ROS detoxification, flavonoids are also translocated toward more internal tissues such as, for instance, the mesophyll [71]. Here, flavonoids exert their role inside or near the target cellular compartments where ROS are generated after severe light exposure, such as the nucleus and the chloroplast [72]. However, flavonoids translocation mechanism to the target organelles is still unknown [64]. In *Phillyrea latifolia*, for instance, *in vivo* observations showed that flavonoids protected the plant against singlet oxygen generated by excessive UV light stress in mesophyll chloroplasts [73]; they can also be transported to the vacuole, where their coupled activity with peroxidases was shown to increase H_2_O_2_ scavenging [74].

Besides radiations, even high temperatures, often combined with drought stress [75], can cause ROS accumulation by affecting evapotranspiration, stomatal closure and by decreasing water content in leaves [76,77]. Flavonoid biosynthesis is an essential trait for plant tolerance and adaptation to extreme temperatures and climatic variations. Plants were shown to activate chemical defense mechanisms by inducing the expression of phenylpropanoid pathway genes (e.g., chalcone isomerase, flavone and flavonol synthase, anthocyanidin synthase), leading to increased concentrations of flavonoids in leaves and photosynthetic tissues [78,79]. The up-regulation of flavonoid biosynthetic genes was seen in wheat and rice, suggesting a role for flavonoids in defense from drought and high temperatures [80,81]. Similarly, in the citrus *Cleopatra mandarin*, which is a highly susceptible species to heat and water stress, a strong antioxidant response was marked by the accumulation of apigenin and polymethoxylated flavones [82]. Furthermore, the response to high temperatures was seen to be stress-specific in tomato (*S. lycopersicum* cv. Boludo); heat stress alone preferentially led to the accumulation of flavonols, and their biosynthesis was reduced under salt stress or under a combination of heat and salt stress, which instead revealed higher concentrations of hydroxycinnamic acids [72].

Excessive soil salinization is often correlated with heat and drought and can cause osmotic stress and toxic ion accumulation leading to ROS formation [22]. Increased concentrations of flavonol glycosides, especially quercetin, isorhamnetin, and kaempferol were indeed observed in *Ginkgo biloba* and in *Casuarina glauca* leaves after treatment with low concentrations of NaCl and helped to improve plant tolerance to the stress [83,84].

Low temperatures during winter can be a source of oxidative stress in plants and therefore cause seasonal fluctuations of flavonoid levels [85]. Sorghum, for instance, is susceptible to frost conditions and responds by accumulating antioxidant molecules such as 3-deoxyanthocyanidin luteolinidin in the roots [86]. To mitigate desiccation stress caused by frost, also the resurrection plant *Haberlea rhodopensis* was seen to accumulate flavonoids and anthocyanins [87]. These observations are consistent with previous studies carried out in *A. thaliana* where increased flavonol content, in particular quercetin and anthocyanin compounds in the over-expressing *pap1-D* line, and the expression of genes involved in flavonoid biosynthesis were proven to be related with cold acclimation, supposedly due to their role as protectors for freezing damages of membranes and proteins. Indeed, most of the flavonoid knock-out mutants showed impaired freezing tolerance or the accumulation of known or unknown flavonoids with potential compensatory effects, highlighting a functional role for these molecules under cold stress [18].

Due to their aromatic structure and the presence of the cathecol group, some flavonoids act as metal ion chelators, therefore avoiding the generation of oxygen radicals and lipid peroxidation induced by phytotoxic concentrations of heavy metals in soil [66,88]. Indeed, flavonoids were seen to form transition complexes with metals in lettuce leaves irrigated with industrial wastewaters polluted by heavy metals such as Pb, Co, Ni and Cd [89], preventing heavy metal ions’ participation in ROS production [54]. An analogous effect was observed in *Erica andevalensis*, a heavy metal-resistant plant that grows in mine soils, showing higher tolerance to Cd due to the defense mechanisms prompted by the flavonoid rutin, among other phenolics [90]. Moreover, the treatment with increasing concentration of copper generated a boost of flavonoid concentration (reaching the order of magnitude of milligrams) in *Belamcanda chinensis* calli, probably to mitigate the impaired expression of the antioxidant enzyme guaiacol peroxidase due to the elevated stress level [91].

The role of flavonoids in plant physiology and homeostasis is not only related to the prevention of oxidative damage but also to other fundamental aspects for plant fitness such as the modulation of auxin transport and the influence on bioavailability of soil nutrients [92,93].

Flavonoids regulate plant morphogenic response to stresses through the modification of the transport of auxins, important phytohormones that coordinate root formation and elongation, phototropic and gravitropic responses [76,94,95]. Numerous studies highlighted that, depending on the substitution groups, flavonoids could exert a negative role in polar auxin transport (PAT), such as kaempferol and quercetin [63,96], or the opposite effect of promoting PAT and root elongation, such as scutellarin [97]. The role of flavonoid in auxin-mediated morphogenic responses was also observed in halotropism, where the accumulation of light-induced flavonoids generated root bending in the perennial grass *Poa trivialis* L. [98], and in cotton axillary buds where decreased flavonoid content generated enhanced auxin efflux and the outgrowth of new shoot branches [99]. Flavonoids appear to directly modulate membrane trafficking of *PIN* proteins, the auxin efflux facilitators: exogenous supply of 1 nM naringenin restored *PIN1* localization in the root tip in the *Arabidopsis tt4* mutant, lacking flavonoid biosynthesis and showing altered auxin transport [100].

The extreme versatility of flavonoids in plant physiology extends to the enhancement of nutrient acquisition in plants due to their involvement in metal chelation and complexation and to their role as reducing agents. Often, essential macro- and micro-nutrients, such as N, P, Fe and Mn, are poorly bioavailable for the plant that, among other strategies, secretes flavonoids to enhance their mobilization and solubility [101]. In fact, aside from their well-known role as chemoattractors and *nod* genes inducers in *Rhizobium*-legume symbiosis [102,103], flavonoid biosynthesis was reported to be upregulated under nitrogen deficiency in tea and tomato plants as a stress response [104,105]. Flavonoid exudation also increased in apple trees subjected to phosphorus depletion, in order to cope with the nutritional stress by prompting either Fe reduction or chelation and consequently P solubilization [106].

## 3. Flavonoids Mediate Plant-Plant Interactions

**Figure 2:**
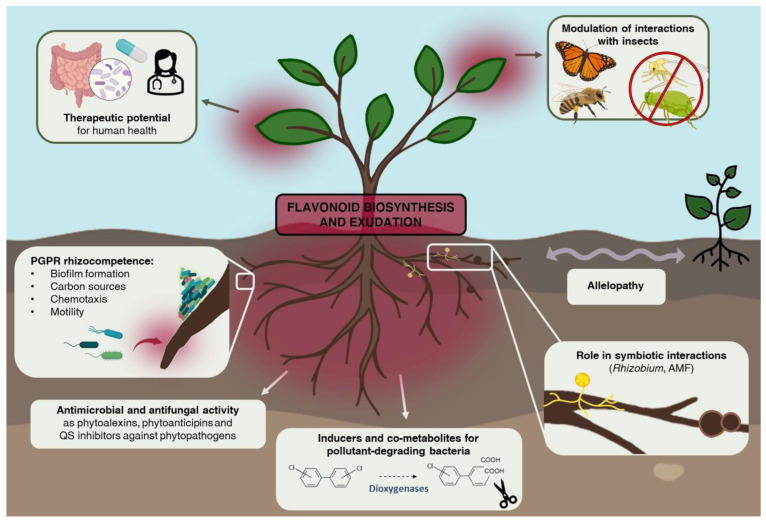
Plant intra- and inter-kingdom interactions mediated by flavonoids. The main aboveground and underground multitrophic interactions, that are orchestrated by flavonoids, are illustrated in the figure. The release of these phytochemicals in the soil modulates allelopathic interactions with neighbor plants and the symbiotic relationship with rhizobia and arbuscular mycorrhizal fungi (AMF). It also mediates rhizocompetence traits of plant growth promoting rhizobacteria (PGPR), exerts antimicrobial and antifungal functions against phytopatogens and stimulates the bacterial catabolism for xenobiotics degradation. Furthermore, flavonoids can tune interactions with beneficial and opportunistic insects. Recently, flavonoids have been proposed as nutraceutical and prebiotics for human health. The image was partially realized using graphic contents from Biorender (https://biorender.com/ (accessed on 2 October 2022)).

Allelopathy is generally used to indicate a phytotoxic interference of a plant on the growth of another plant belonging to the same species (autotoxicity) or to a different one (allelopathy *in sensu stricto*), in the same soil niche [107] (Figure 2).

The process is mediated by a complex network of biochemical signals, due to the release of root or leaf exudates by the allelopathic plant and volatile molecules or leachate from plant litter decomposition. In soil, these allelochemicals range from 10^−5^ to 10^−6^ M [108] and, despite these low concentrations, they lead to the delay and/or suppression of germination, growth and development of neighboring sensitive plants [23]. As an example, the invasive tree black cherry (*Prunus serotina*) releases seasons-variable phytotoxic compounds from the leaves. Among them, the monoterpene linalool did not show a germination inhibitory effect but affected the root length and the seedling growth of pine plantlets in European forests [109]. Several reports indicated that young seedlings are more prone to the phytotoxic effect of allelochemicals rather than germinating seeds, as observed also for radish and maize plantlets exposed in soil to ground leaves of *Fallopia japonica* and *bohemia*, one of the most invasive species in Europe [110].

Perennial legumes, like clover and alfalfa, showed both autotoxic and allelopathic features [111] that were speculated as arising from their evolutionary establishment in extremely dry and nutrient-poor Mediterranean soils. Under these adverse conditions, plants adopted allelopathic strategies to improve their survival chances by preventing the growth of young seedlings near mature plants [112]. The alfalfa saponin medicagenic acid, assayed at 500 ppm, exerted strong phytotoxicity on germination and seedling growth. The knowledge of this autotoxicity has an influence on agronomical decisions on alfalfa replanting in fields in which alfalfa allelochemicals of previous plantation could persist [113]. Furthermore, allelopathy explains weeds fitness in invasive ranges, enhancing the competitive ability against native plant species, competing for resources, light, space, and nutrients [114,115]. It is estimated that weeds contribute to 28% reduction of crop yield [116], with severe economic losses [117].

The role of flavonoids as allelopathic molecules has been largely acknowledged [118,119,120]. Often, unique compounds are specifically synthesized, as in the case of the barley-produced saponarin, a newly identified flavonoid, to suppress *Bromus diandricus* spread [121]. According to the “novel weapon hypothesis” flavonoids accumulation may drive the establishment success of invasive plants and, in particular, allelopathic actions exerted specifically by molecules belonging to the flavonols family may represent a signature of tree-invasive species [122].

Recently, allelopathy was exploited to develop agronomic practices for weed integrated management by planting allelopathic crops, thus reducing the use of agrochemicals [123,124], or by using identified flavonoid allelochemicals, such as coumarin, and bioherbicides [113]. Coumarin supplements (10 µM) can significantly alter *Arabidopsis* root architecture, by reducing up to 50% primary root length and significantly increasing the number of lateral roots [125]. Modifications in root anatomy are a well-described mechanism in allelopathic responses: exposition to allelochemicals can trigger toxic responses mediated by proteolytic activities and eventually culminate in organ and tissue cell death, thus affecting plant growth [107].

To develop sustainable bio-herbicides, allelopathic plants may be used as a source of phytochemicals to cope with weed invasiveness. Methanolic extracts (20%) of *Artemisia santolinifolia*, for instance, were shown to cause severe oxidative stress and permanent wilting in major weeds, and this effect seemed to be due to a higher content of phenols and flavonoids (rutin and quercetin) in plant extracts [126].

In Africa, smallholder farmers cultivate *Desmodium uncinatum* as intercropping plant to prevent the attachment and germination of the parasitic weed *Striga* [127], an effect that was found to be mediated by several (iso)flavonoid glycosides released in the legume root exudates [128]. In this case, flavonoids orchestrated a complex network of biological events that culminated in *Striga* suicidal germination, whereas uncinanone B promoted weed seed germination, uncinanone C and several di-C-glycosylflavones prevented the propagule attachment to host roots [129].

The allelopathic efficacy seems to rely on the concentration [130] and persistence of allelochemicals in soil water solution, that is affected by environmental factors, soil edaphic properties and plant-related growth stages [131]. White clover stands release at relatively high concentration different aglycones such as formononetin (~4500 μmol/kg plant dry weight), medicarpin (~950 μmol/kg plant dry weight), and kaempferol with phytoinhibitory activity and this latter, among others, persisted for days in field soil (~1200 nmol/kg dry soil) [132]. Similarly, allelopathic rice cultivars accumulated two flavone glycosides in root tissues and, once exudated, they were rapidly converted in aglycone forms, more resistant to microbial degradation and less mobile in paddy soil, thus enhancing their suppressive effect against rice paddies-infesting weeds such as *Echinochloa crus-galli* [133].

Flavonoids’ mechanism of action in allelopathy is far from clear [63], although it is believed to be related to the interference with ATP production and to auxin transport and degradation, resulting in dramatic alteration of the root morphogenic program of target plants [134]. Exposition of *Arabidopsis* roots to 4 mg/mL of the invasive *Conyza canadensis* plant extract led a decrease in root tip vitality and a ROS burst, coupled to the induction of stress-responsive genes, pathways and detoxifying machinery [135]. The death of the root system of neighboring plants was observed in knapweed *Centaurea maculosa*-infested fields and this effect was caused by the dramatic wave of ROS induced by the (-)-catechin released by the weed [136], revealing a potential pro-oxidant function of flavonoids in allelopathic interactions [137]. Administration of (-)-catechin triggered programmed cell death that expanded from the root region to the stele, presumably by eliciting a ROS-induced calcium wave that alters cell ionic homeostasis, unbalancing cellular pH [136].

## 4. Flavonoids as Weapons against Phytopathogenic Attacks

**Figure 3:**
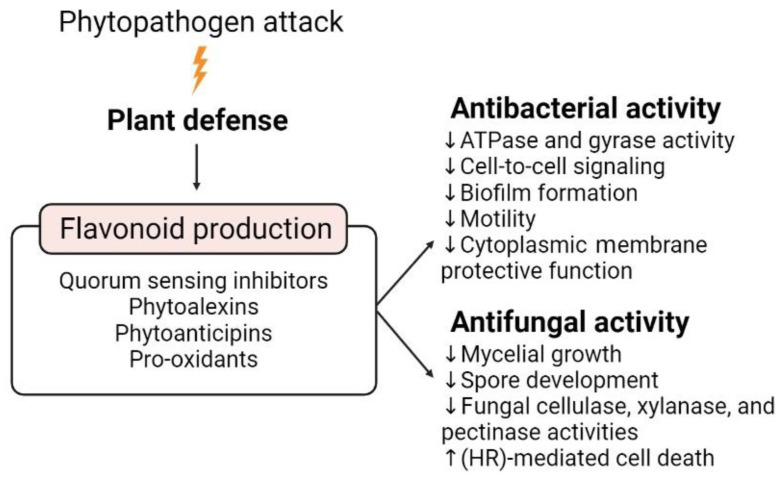
Flavonoid-mediated plant defense strategies against biotic stress caused by phytopathogen attacks. The attack by microbial phytopathogens induces plant defense responses (represented by the thunder symbol), including an enhanced flavonoid production and exudation. Flavonoids play numerous roles in plant defense against biotic stresses: they are quorum-sensing inhibitors, pro-oxidants and some of them are defined as phytoalexins and phytoanticipins defense compounds. These features confer flavonoids with antibacterial and antifungal activities that are mediated by a diverse array of mechanisms, some of which are reported in the figure. The down-oriented arrow (↓) represents an inhibitory effect, whereas the up-oriented arrow (↑) represents an induction effect.

Plants and their associated microorganisms coevolved as a unique meta-organism defined as the plant holobiont [138,139]. The interplay between plant and microorganisms, in particular those colonizing the rhizosphere and the endosphere, is specifically mediated by exuded metabolites and chemical signals exchanged as a form of communication and essential in maintaining the holobiont health status and performance [140]. Flavonoids constitute a conspicuous percentage of root-exuded secondary metabolites and are among the most studied molecules involved in plant-microorganism interactions [33,36] (Figure 2). Flavonoids play a crucial role in orchestrating plant defense upon phytopathogen attacks since they can interfere with the mechanisms that drive bacterial and fungal virulence [141,142]: they are antimicrobial and antifungal agents and act as priming agents of plant defense responses to react rapidly upon infection perception [142,143] (Figure 3).

Indeed, they can act as quorum-sensing inhibitors (QSI) by multiple mechanisms, including the reduction of signal molecules biosynthesis, the inhibition of cell-to-cell signaling and biofilm formation, and the competition for receptor binding sites by mimicking signal molecules [144,145]. Naringenin and quercetin, for instance, interfere with QS by downregulating the expression of genes involved in the production of acyl-homoserine lactones, typical signal molecules during *P. aeruginosa* infections [146,147,148] and by hindering the correct functioning of QS receptors, specifically due to the presence of two -OH groups on the flavonoid A-ring. This specific molecular structure has an antagonist role toward pathogen autoinducers (AIs), compounds that coordinate the expression of genes for virulence and survival and prevent DNA binding of the AIs-receptor complexes, hence down-regulating the expression of pathogenic factors [149]. Another example of QS inhibition observed in a phytopathogenic strain was the suppression of the QS-regulated toxoflavin production in *Burkholderia glumae*, causal agent of rice grain rot and wilt, mediated by two apigenin derivatives [150,151]. By using a *B. glumae* biosensor strain, engineered to trigger β-galactosidase activity upon toxoflavin expression [152], it was observed that the inhibitory activity of the apigenin derivatives on the strain occurred at low concentrations, 6.76 and 7.87 µM respectively [150].

Other exemplary flavonoid antimicrobials were found in the root exudates of *Scutellaria baicalensis,* namely wogonin and baicalein. The former was effective *in vivo* in plants infected by the rice fungal pathogen *Magnaporthe oryzae* at the concentration of 500 µg/mL, whereas the latter showed inhibitory effects *in vitro* against phytopathogenic bacteria such as *Acidovorax avenae* and *Ralstonia solanacearum* at MIC 19 µg/mL (MIC, minimum inhibitory concentration) [153]. Furthermore, the enhanced production of phenolics, including quercetin-3-*O*-glucoside, explained a cultivar-dependent resistance against *Plasmopara viticola* in grapevine compared to a sensitive cultivar [154]. Quercetin-3-galactoside is also able to counteract *in vitro Botrytis cinerea* pathogenic traits by reducing spore germination at the early phase of infection and by inhibiting germ tube elongation, thus potentially reducing the pathogen penetration ability [155].

The role of flavonoids in sustaining plant defense is also expressed by their action as (i) phytoalexins, namely *de novo*-synthesized and pathogen-elicited defense molecules with antibacterial and antifungal activity, and (ii) as phytoanticipins, constitutive molecules that are produced and accumulated before pathogen exposure, that can be rapidly employed by the plant when needing a swift defense response [156,157]. Isoflavonoids and their derivative pterocarpans, such as medicarpin and pisatin, produced respectively by alfalfa and pea plants, are known phytoalexins, active against fungal pathogens as inhibitors of mycelial growth or inducers of hypersensitive response (HR)-mediated cell death [158,159,160]. Conversely, flavonols showed a putative phytoanticipin role in mildew-infected blackberry [161]: despite a low concentration in infected plants, they can safeguard protection from the pathogen. This is explained by the fact that, upon pathogen infection, pre-existing phytoanticipin flavonoids can be transformed into active phytoalexins [161,162]. Some exemplary cases are daidzein, a soy phytoanticipin that, once elicited by pathogenic microbes, is transformed by prenylation into glyceollin pterocarpans [163]. Similarly, the active aglycone form of maackiain is obtained by hydrolysis of the conjugated compound and released by infected red clover plants [164].

Flavonoid pro-oxidant activity represents a further weapon against phytopathogens [165]: pretreatment with 250 µg/mL quercetin [166] or 100 µM naringenin [167] induced H_2_O_2_ production in *Arabidopsis* plants, causing oxidative burst and callose deposition in host cells, magnifying the defense response against *Pseudomonas syringae* pv. tomato DC3000. The addition of catalase or of glutathione caused the reverse effect to that observed with flavonoid pretreatments, making the plant more susceptible and therefore implying the need for H_2_O_2_ accumulation in plant resistance to *P. syringae* infection [166]. However, the mechanisms through which flavonoid pro-oxidant activity can increase plant resistance to pathogens have not yet been clarified and further investigations are needed to unveil their correlation with plant defense responses.

## 5. Flavonoid Exudation Affects the Structure and Function of Root-Associated Microbiomes

Flavonoids interactions with rhizosphere/endosphere microbiomes are efficient means exploited by the plant to communicate with soil microorganisms and to recruit beneficial plant growth-promoting (PGP) bacteria [6,168]. These bacteria largely contribute to the holobiont fitness by alleviating nutritional shortages, by producing phytohormones and volatile organic compounds to enhance plant development [169,170,171] and by repressing attacks from plant pathogens acting as biocontrol agents [38,172]. One of the most studied interactions between plants and beneficial microbes is mycorrhization, where arbuscular mycorrhizal fungi (AMF) colonize roots and improve plant fitness by alleviating biotic and abiotic stress factors [173]. The role of flavonoids in mycorrhizal symbioses is not only associated to the enhancement of hyphal growth and spore germination [32,173]; interestingly, mycorrhiza helper bacteria (MHB), closely associated to specific AMF, can produce Nod factors and induce root exudation of flavonoids that act as signals to attract AMF, thus facilitating root colonization [174,175].

In general, PGP activities are related to the ability of bacteria to colonize and persist on plant roots, which is defined as rhizocompetence [176,177,178] (Table 1). An important feature in rhizocompetence is possessing the catabolic enzymes to use root exudates like flavonoids as nutritional sources, outcompeting other bacterial species [179,180] (Figure 2). Some beneficial soil bacteria belonging to the genera *Bacillus* and *Pseudomonas* and endophytic fungi like *Paraconiothyrium variabile*, for example, are known to effectively metabolize flavonoids [6,181]. The strain *Pseudomonas putida* PLM2 was seen to degrade quercetin via aerobic dehydroxylation, but it could also use other flavonoids such as naringin, naringenin, daidzein and apigenin as carbon sources [182]. Other flavonoid-influenced rhizocompetence traits are bacterial motility, chemotaxis toward root exudates and biofilm formation for accommodation on root surfaces [183,184]. Naringenin-mediated chemotaxis of *Aeromonas* sp. H1 toward *Arabidopsis* roots, marked also by the over-expression of genes for flagellar biosynthesis, was essential to protect plants from the deleterious effect of drought stress [185]. Similarly, phloretin and apigenin enhanced *Pseudomonas fluorescens* 2P24 growth and swarming motility *in vitro* [37]. Biofilm formation was enhanced by rutin in *Bacillus subtilis* [186] and by apigenin in the N_2_-fixing bacterium *Gluconacetobacter diazotrophicus*, both associated to rice plants, thus potentially improving plant growth and defense from pathogens through an indirect mechanism [187].

Concomitantly, plant colonization by PGP bacteria was shown to alter plant flavonoid production, especially under stress conditions [196]. Indeed, a consortium of three PGP microorganisms, including a *Pseudomonas* strain, *Mesorhizobium* sp. and *Trichoderma* enhanced H_2_O_2_ synthesis in *Cicer arietinum* to counteract *Sclerotium rolfsii* attack. In parallel, the beneficial consortium increased accumulation of the flavonols quercetin, myricetin and kaempferol in chickpea plants to protect plant cell membrane from oxidative modifications [197]. Similar increase in total flavonoids content was reported in maize plants challenged by high salinity stress [198,199] and in drought-exposed pennyroyal [200] treated with beneficial bacteria.

**Figure 4:**
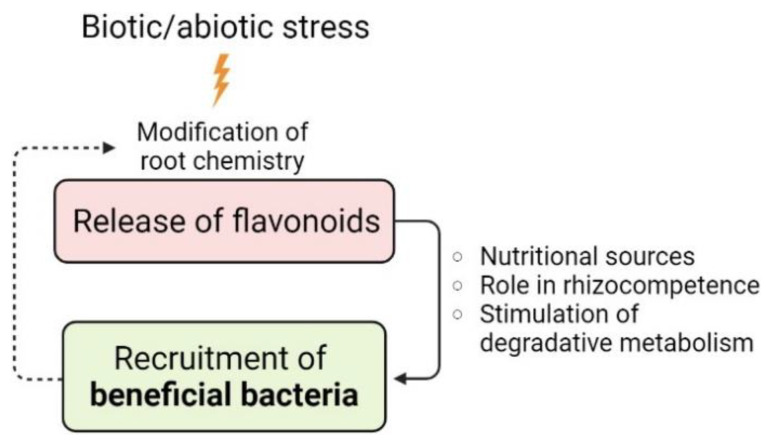
Role of flavonoids in the plant “cry-for-help” strategy to cope with environmental stresses. Modification of root chemistry is part of the physiological responses that plants adopt to cope with environmental stresses (represented by the thunder symbol). This phenomenon is known as “cry-for-help” and it induces, among others, the exudation of flavonoids in the rhizosphere. These phytochemicals play multiple roles in the recruitment of soil microorganisms, which can establish beneficial interactions with the host plant and contribute to the holobiont fitness, mitigating the stress deleterious effects. Flavonoids can be exploited as nutrients, induce efficient colonization of the root system and act as inducers for the expression of catabolic genes for the degradation of xenobiotic compounds. In turn, beneficial microbes colonizing the rhizosphere and the endosphere can affect the root exudation pattern (dashed arrow), favoring the establishment of suitable conditions for their growth in these niches.

It was hypothesized that, when subjected to biotic and abiotic stress conditions, the plant exudates secondary metabolites to actively recruit beneficial microorganisms in order to respond and minimize the stress [86], exploiting a mechanism known as “cry-for-help” [201,202] (Figure 4).

**Figure 5:**
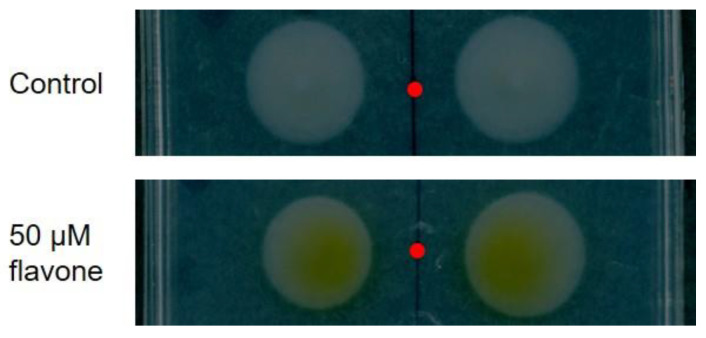
Flavonoids-mediated enhancement of the PCB degrading metabolism. The PCB-degrading strain *Pseudomonas* strain JAB1 was placed in close contact with flavone, which is able to induce its degradative metabolism [211] as visible by the yellow coloration assumed by the grown colonies, possibly due to the presence of a degradation intermediate [212]. The red dots represent the sites in which the solvent (control) or the flavonoid flavone were plated, according to the protocol for chemotaxis described by Reyes-Darias and colleagues [213]. Picure by Elisa Ghitti and Eleonora Rolli.

This rescue strategy is crucial when plants are exposed to phytotoxic xenobiotics such as, for instance, polychlorinated biphenyls [203]. Under these circumstances, plants tune their exudation profile to favor the recruitment of microbes endowed with the degrading enzymatic machinery to transform recalcitrant substances in more bioavailable and less phytotoxic compounds [204,205]. An increasing body of evidence indicates that flavonoids, due to a similarity in the chemical structure, are crucial in stimulating the microbial-degrading metabolism of PCBs (Figure 5) by acting as growth substrates, co-metabolites or inducers of the *bph* operon [206,207,208], that comprises the catabolic genes for biphenyl aerobic oxidation and encodes for the dioxygenases necessary for its mineralization [209,210].

Therefore, the flavonoid-mediated plant-microbe crosstalk paves the way for rhizoremediation approaches for polluted soils clean-up [40,214]. The PCB-degrading bacterium *Rhodococcus erythropolis* U23A, grown co-metabolically with a different combination of carbon sources and flavonoids such as flavanone, flavone and isoflavone, showed the ability to degrade 4-chlorobiphenyl to 4-chlorobenzoate with a higher efficiency than in the presence of biphenyl [189]. This effect was directly dependent on the flavonoid-driven induction of *bph* genes [215], a feature observed also with flavanone and catechin in *Pseudomonas alcaliphila* JAB1 [211]. A microcosm experiment revealed that the addition of naringin to the soil had an impact on bacterial community structure, promoting the growth of *Burkholderia* spp., and led to an enhanced degradation of numerous PCB congeners [216].

Flavonoids exert similar functions on another wide class of pollutants, namely polycyclic aromatic hydrocarbons (PAH): PAH-degrading bacteria isolated from cucurbits used flavonoids like morin as unique carbon sources, suggesting a relation in the metabolic pathways and the putative promotion of PAH degradation induced by flavonoids [217].

A deeper understanding of the mechanisms involved in beneficial plant-bacteria interactions mediated by flavonoids may be crucial in implementing sustainable strategies aimed at increasing plant growth and productivity [36], accelerating phyto-rhizoremediation [218], improving plant protection from pathogens and, more generally, the holobiont resistance to stresses [219].

## 6. Flavonoids Modulate Behavior and Life History Traits of Insect Herbivores, Natural Enemies and Pollinators

Flavonoids are known to modulate plant interactions with insects, including herbivores, natural enemies (i.e., predators and parasitoids) and pollinators [220,221] (Figure 2). Together with other plant pigments (e.g., betalains and carotenoids), these phytochemicals confer color, fragrance and taste to flowers, fruits and seeds, attracting not only insects, but also birds and mammals, which provide important ecosystem services *i.e.*, pollination and seed/fruit dispersal [222]. For instance, quercetin preference by the honeybee *Apis mellifera* has been verified in free-flight semi-field assays [223]. Mutations in the flavonoid biosynthetic pathway showed a shift of pollinators’ preference [224]. Loss-of-function mutations of the transcriptional factor *ANTHOCYANIN2* (*AN2*) in *Petunia axillaris* resulted, indeed, in white-colored and moth-visited flowers, whereas *P. integrifolia* flowers with functional *AN2* alleles harbored anthocyanin-colored petals and were efficiently pollinated by bees and butterflies [224].

Flavonoids have a crucial role in plant protection and defense against phytopathogenic insects by modifying the plant palatability, reducing its digestibility, decreasing its nutritional value, but also attracting useful predators of insect herbivores. They can behave as toxins, binding the insect digestive enzymes, and thus interfere with the host development, reproduction and molting, affecting ultimately the fitness. They can inhibit ecdysone-dependent pathway and acetylcholinesterase, impairing molting, or hinder the activity of glutathione S-transferases enzymes, involved in insecticide detoxification [50,63,225]. High flavonoid levels in tomato reduced the plant attractiveness and the feeding activity of the whitefly *Bemisia tabaci*, which in turn decreased the spread of the whitefly-transmitted virus responsible for an economically important disease (*i.e.,* tomato yellow leaf curl disease, TYLCD) [226]. Feeding tests verified the repellence or feeding deterrent effect of different flavonoids in several insect and nematode species [227,228], supporting their use as biopesticides as alternatives to traditional synthetic molecules [229]. Quercetin, for instance, is a repellent and insecticidal compound for different plant-feeder insects like aphids [230]. However, its ingestion activates detoxification enzymes in useful insects like honeybees, extending their longevity and enhancing pesticide tolerance, hence highlighting a beneficial role of quercetin for pollinators [231,232]. In this context, an interesting perspective relies on the use of agricultural wastes as flavonoids’ source material under the frame of a circular economy approach, once field experiments and evaluations on features such as environmental fate and target specificity would have ensured their safe use [229]. Flavonoids can have indeed a harmful effect on nontarget beneficial species, as observed on the zoophytophagous generalist predator *Orius sauteri*, a biological control agent of whiteflies, which showed a reduced oviposition, nymphal survival, and development on flavonoids-producing tomatoes [233]. Several studies also reported the phagostimulant or neutral activity of flavonoids in dependence of the insect species considered and of the molecule and dosage selected [228,234]. For instance, quercetin and pinocembrin at low concentration (0.01–1 µg/cm^2^) have phagostimulant effects on *S. frugiperda* on treated lettuce foliage [228], whereas at higher concentrations (10, 50, 100 µg/cm^2^) the effects of these two molecules were different. Pinocembrin showed an antifeeding effect for all the high concentrations, whereas quercetin had a neutral activity at concentrations of 10 and 50 µg/cm^2^, becoming antifeedant at a concentration of 100 µg/cm^2^ (although with lower effects than pinocembrin) [228]. A recent review summarizes a literature search on insect-quercetin interactions to suggest the use of this flavanol to reduce herbivory without damaging natural enemies and pollinators: harmful and nonharmful effects were reported on different insect species, considering different application dosages and behavior and life history traits [230]. However, authors highlighted the need for field experiments to substantiate the results [230].

Flavonoids can also regulate insect oviposition [63]. Quercetin glycosides are oviposition cues for the monarch butterflies [235], whereas quercetin, taxifolin, and naringenin (the last one to a lesser extent) can stimulate oviposition in the predatory ladybird beetle *Coleomegilla maculata* [236]. Interestingly, quercetin can also orient *C. maculata* to oviposition sites when conspecifics are present, underlining the possible exploitation of this phytochemical in mass-rearing facilities [237]. Conversely, oviposition can be negatively influenced by the presence of flavonoids [230]. Flavone, rutin, quercetin, myricetin, fisetin, quercitrin, and partially purified flavonoids from *Calotropis procera* (a popular medicinal plant of Africa and Asia tropics), reduced egg laying of the adzuki bean weevil *Callosobruchus chinensis* in a dose-dependent manner [238]. Quercetin also deterred oviposition of the melon fruit fly *Bactrocera cucurbitae* and its presence on test substrates could outcome in a lower number of ovipunctures under multiple-choice conditions, as also exerted by rutin [239]. A decrease in flavonoid content in tomato infested by *B. tabaci* positively modulated the oviposition preference of the insect [240].

Even if many efforts have been performed to elucidate insect-flavonoid interactions, also focusing on specific molecules [220,230], future in-depth work should consider more insect species and different flavonoid compounds, considering a multitrophic level of investigation (*i.e.*, among plant, herbivore, natural enemy, pollinator, and generally nontarget species) and clarifying the molecular mechanisms that underlie these interactions.

## 7. Flavonoids as Nutraceuticals and Prebiotics for Human Health

Flavonoids and their metabolites can improve human health by acting as phytochemicals [241], nonessential molecules consumed through food or dietary complements, that modulate signaling pathways and decrease inflammation, immune response and oxidative stress processes [43]. This review will treat this topic highlighting flavonoids’ role as inter-kingdom signaling molecules also in the context of human pathologies: other works have dedicated valuable efforts to elucidate the medical and nutraceutical potential of these compounds [41,242,243,244,245,246].

Flavonoids were shown to exert a variety of nutraceutical features such as antioxidative, anti-diabetic, anti-proliferative, anti-carcinogenic, anti-microbial and immunomodulatory abilities [44]. Due to these abilities, flavonoid supplements have been largely investigated in studies for prevention or drug efficacy evaluation in several pathologies including cancer, atherosclerosis, stroke, neuroinflammatory diseases, age-related neurodegeneration, diabetes, human herpes virus (HHV) infections and recently also COVID-19 respiratory disease [43,60,247,248] (Figure 2). Interestingly, one of the main effects of these illnesses is oxidative stress, which induces harmful damages on membranes, lipids, proteins, lipoproteins and DNA in humans [249], similar to the dangerous effects previously described in plants. The role of flavonoids as conventional hydrogen-donating antioxidants has been extensively reviewed and leads to a paradigm shift in their acknowledged functions in human disorders [43]. According to an emerging scenario, these phytochemicals may exert their beneficial activities in human health mainly by interfering with signaling pathways [242].

Diet is the main source of flavonoids for humans, since they are common constituents of fruits, vegetables and legumes [250]. Berries are known to contain a wide range of antioxidant flavonoids as anthocyanidins, the flavone luteolin and numerous flavonols [251]. Isoflavonoids, like genistein and daidzein, are the main class present in legumes due to their role in nodule formation in the symbioses with rhizobia [252], and flavanones as hesperidin and naringenin can be found in citrus species and grapes [253,254,255]. Flavonoids are also present in fruit and vegetable food wastes, making them profitable resources to obtain useful biomolecules for the formulation of functional foods and nutraceuticals in a circular economy approach [256]. Fourteen different flavonoids have been identified in tomato seeds extract, including quercetin, kaempferol and isorhamnetin glucosides [257], and rutin and naringenin were found to be the main constituents of total phenolics in tomato peel fiber [258].

Once introduced through the diet, flavonoids are absorbed and digested in the intestine by enzymes expressed by intestinal epithelial cells and by the gut microbiota, giving rise to more absorbable aglycones and a mixture of smaller phenolics with varying degrees of hydroxylation, glucuronidation, sulfation and methylation [259,260]. Parent flavonoids and their metabolic products can exert their biological function locally in the gastrointestinal tract or can pass to the systemic circulation to reach the peripheral body districts where they perform systemically beneficial actions [261]. At the intestine level flavonoids and their metabolites exert a prebiotic effect for the host’s immune system and gut health [244]. Through an unknown mechanism, dietary flavonoids can support the growth of probiotic bacteria such as *Bifidobacteriaceae* and *Lactobacillaceae* and inhibit pathogens such as *E*. *coli* and *Helicobacter pylori*, thus improving gut health and immune modulation by reducing endotoxin production, sustaining nutrients absorption and intestinal barrier functionality [262,263]. Presumably by inhibiting the gyrase, a topoisomerase involved in bacterial DNA replication [264], 50 µg/mL quercetin was found to inhibit the growth of *Ruminococcus gauvreauii* [265], a gallbladder bile-inhabiting bacterium [266] whose abundance has been associated to altered gut composition in patients affected by coronary artery disease [267]. Depending on their structure and dose, flavonoids can affect the structure of the gut microbiome, by stimulating the abundance of specific genera [42], providing an analogy to the mechanism driving the plant microbiome structuring. Rutin and quercetin supplements at 100 µg/mL stimulated the growth *in vitro* of *Bifidobacterium bifidum*, although at the same concentration quercetin and hesperidin inhibited the growth of *Bifidobacterium adolescentis*, showing contrasting effects depending also on bacterial species and strains [268].

Furthermore, flavonoids can perform other beneficial roles in the gastrointestinal tract. Indeed, diets rich in specific flavonoid families such as anthocyanidins, flavonols, flavones, and isoflavones showed a positive trend for a decreased risk in developing colorectal cancer [269], presumably by inhibiting pro-oncogenic signaling pathways [270].

At a systemic level in the body districts, apigenin, quercetin, chrysin, luteolin, kaempferol and fisetin are flavonoids described for their beneficial role in human health and their mode of action seems to rely on the suppression of inflammatory markers such as interleukins and down-regulation of inflammatory pathways mediators such as NF-kB, that responds early to harmful cellular stimuli, and MAPK cascades, including p38-, JNK-, and ERK-mediated signaling [41]. Myricetin is an isoflavonoid present in different edible parts of the plant and in fruits such as oranges, berries and teas [271]. Several *in vitro* studies highlighted myricetin as a potential chemotherapeutic agent able to interfere with dysregulated signaling pathways in cancer progression, invasion and metastasis by regulating ER stress, NF-kB, mTOR and telomerase [272]. In human papillary thyroid cancer, 100 µM myricetin treatments induced cytotoxicity and DNA condensation, finally causing cell death of SNU-790 cells by disrupting the mitochondrial membrane potential and regulating caspase cascades [273]. Myricetin-mediated cytotoxicity was observed in triple-negative breast cancer cells due to oxidative stress: autoxidation of 50 µM myricetin in the growth medium led to extracellular H_2_O_2_ formation and triggered intracellular ROS production, ultimately causing DNA damage [274]. Naringenin, as well, has been demonstrated to be effective against a wide range of tumor types by suppressing cancer progression programs [275]. Genistein, an isoflavonoid present in soy-derived foods, was hypothesized to reduce SARS-CoV-2 infectivity by inhibiting the virus strategy for entrance in human epithelium [276]. Numerous *in vitro* studies also highlighted the role of flavonoids in the treatment of HHVs infections and in preventing the associated neurological diseases [60]. Flavonoids isolated from *Morus alba* L. showed antiviral activities toward the widespread herpes simplex virus type 1 (HSV-1) through different mechanisms that led to inhibited viral proliferation: morusin interfered with the expression of the glycoprotein necessary for cellular binding and infection and reduced the virus-induced ROS content [277], and kuwanon C, T, U, and E hindered HSV-1 replication machinery [278].

Despite being under the spotlight for their wide potentials in therapeutic use, flavonoid administration still lacks *in vivo*, preclinical and clinical studies for their validation in the treatment of human diseases. There is, therefore, a huge need for clinical research to assess flavonoids’ efficacy in human health. Furthermore, the elaboration of uniform guidelines in the design and reporting of flavonoid studies is advocated, in order to translate clinical evidence and research findings in valuable recommendations for patients [279]. In a pharmacological perspective, it will be crucial to understand the structure-activity relationship, considering that several flavonoids exert diversified, multiple effects on different pathways. Thus, a precision medicine approach is necessary to identify the mechanisms of action, eventual off-target effects, investigate the most active flavonoid molecules, and use them in combination therapies. This knowledge will be essential to understand the significant inter-individual variability observed in dietary flavonoid benefit supplementations [280].

So far, flavonoids are extracted from natural plant resources through separation techniques [281,282]. Recently, much progress has been made through a synthetic chemistry approach to investigate the structure-activity of flavonoid moieties [283], to promote synthetic units that control a precise interaction site within the biological system [284,285], and to modify the flavonoid backbone to achieve new functionalities [286], paving the way toward the discovery of new drugs [287].

A major drawback in flavonoid medical application is linked to their poor bioavailability and low solubility in oral formulations, which makes it necessary to develop valuable delivery systems to the target cells [272]. Nanocarriers may solve this issue, by protecting encapsulated flavonoids from oxidation or degradation, and increasing their stability, solubility and bioavailability to the target tissue [44,288]. A diverse set of nano-drug delivery systems (lipidic, polymeric, micelles, nanoemulsions and nanoparticles) has been developed for naringenin encapsulation to overcome its medical application issues due to the molecule instability and low bioavailability [289,290].

## 8. Concluding Remarks

Although their primary ancestral role in evolution seems to be correlated with protection against the excess of radiant energy, flavonoid biosynthesis is triggered by a broader array of abiotic stresses and their main function is to cope with stress-induced ROS oxidative burst. Despite obvious differences between plants and animals in stress responses, ROS trigger very conserved signaling cascades and induce common damages in both systems [291,292], explaining the wide ability of flavonoids to act as ROS scavengers in plants and to modulate inflammation-signaling cascades in animal and human *in vitro* models [43].

Besides ROS protection, flavonoids showed additional beneficial functions, such as, (i) the influence on phytohormone homeostasis and stress-induced morphogenesis, which contribute to tune the plant plastic response to adverse environmental conditions [293], (ii) the soil detoxification from xenobiotics, and (iii) the microbiome modeling in the “cry-for-help” mechanism [204]. These metabolites can act also as signaling molecules, endowing plants with the ability to perceive the environment and to communicate with the biotic components belonging to other species, genera, families and kingdoms [36]. These multi-kingdom communication dynamics affect high biodiversity hotspots such as the rhizosphere as well as the human gut, where dietary flavonoids are metabolized [294].

Although plant secondary metabolites have been described for their ability to perform activities across different kingdoms such as, for instance, plant defense against weeds and pathogens [295], so far only flavonoids have shown such a large variety of functionalities and conserved beneficial effects in plant, animal and human systems and the ability to mediate interactions with prokaryotic and eukaryotic cells.

In the vision of phytochemicals application in agriculture to sustain crop growth, phytoprotection and yields, flavonoids are élite molecules. Quercetin, for instance, is a potent flavonoid with a diverse set of functions in plants [225]. Preliminary pot experiments suggest its potential as biofertilizer: spray inoculation of quercetin on wheat leaves increased physiological parameters related to chlorophyll content and fluorescence, and induced the accumulation of phenolics that are correlated with increased resistance to abiotic stress in a dose-dependent manner [296]. Seed priming with quercetin alleviated chromium toxicity in *Trigonella* seedlings, enhancing flavonoids accumulation in plant tissues, potentially quenching ROS induced by the metal contamination [297]. Another promising flavonoid for field application is naringenin, although its involvement in plant defense is poorly elucidated. A preliminary study in tobacco highlights that naringenin is endowed with a low-medium antifungal activity against *Phytophtora nicotianae*, although it induces the expression of salicylic acid responses to counteract the pathogen attack [298].

A main issue for flavonoid applications in agriculture is their stability in soil that depends, among other factors, on the specific molecule and the degradative activity of soil microbiomes. Flavonoids are considered rather stable compounds, although apigenin and kaempferol half-life in soil is estimated within only 4 and 14 days respectively. Nevertheless, these lifetimes of permanence in soil are limiting factors for flavonoid exploitation as biofertilizers or biopesticides because of the requirement for more frequent applications to maintain these phytochemicals at effective concentrations. The adoption of delivery systems based on encapsulation could contribute to increase flavonoid stability and a sustained release in soil. Nanoliposomes were adopted to encapsulate quercetin to efficiently target the phytochemical inside leaf cells through osmosis or endocytosis, overcoming its low solubility which is considered as an important factor limiting the phytopathogen control effect. Nanoliposome-quercetin, indeed, could inhibit the expression of plant hsp70 proteins that are generally induced under stress and which are host factors hijacked by viruses to assemble their replication complex, thus acting as an outperforming antiviral agent even under field conditions [299]. On the other hand, soil-dissolved organic carbon (DOC) strongly reduces flavonoids’ lifetime in soil. It is estimated that ~70% of flavonoid signaling potential is attenuated by sequestration to DOC through a metal-mediated oxidative reaction that leads to dimerization [300]. This phenomenon occurs with a variety of plant sources and mainly implicates Mn^3+^, a metal that is abundant in litter decomposition [300,301]. Flavonoids are also degraded aerobically by diverse soil microbes through monooxygenase enzymatic activities, as observed in *Herbaspirillum seropedicae* mutated in the *fdeE* gene which lost the capacity to use naringenin as a carbon source [302]. This knowledge could be the picklock to design a holobiont-metabolic network utilizing plant with a specific flavonoid exudation profile [303] coupled to information about the lifespan of these metabolites under different soil conditions. This approach would pave the way to engineer beneficial plant-microbe interactions, reduce phytopathogen outgrowth and propose effective soil amendments [24]. In this vision, further research is needed to increase knowledge about flavonoid bioactivity toward microorganisms, their concentration of use, environmental fate, degradative kinetics and influence on soil microbiota. These data are crucial for an environmentally safe application of flavonoids in agricultural practices. A similar concern arises also for flavonoids utilization in medical treatments. Despite mounting evidence on their protective role in several human pathologies, basic research and clinical trials are needed to assess, respectively, their mechanism of action and their efficacy and safety for drug development.

## Figures and Tables

**Table 1:** Impact of flavonoids on growth and rhizocompetence traits of plant growth-promoting bacteria (PGPB) through *in vitro* experiments.

Bacteria	Rhizocompetence Traits	Flavonoids	Concentration	Main Effects	Reference
*Aeromonas* sp. H1	Chemotaxis Biofilm formation	Naringenin, kaempferol, quercetin	100 µM	Induction of chemotactic movement Biofilm formation Up-regulation of flagellar biosynthetic genes	[185]
*Gluconacetobacter* *diazotrophicus*	Biofilm formation	Apigenin	20–100 µM	Dose-dependent induction of biofilm formation, without impacting bacterial growth	[187]
*Bacillus licheniformis*, *Bacillus subtilis*, *Acinetobacter junii* Pb1	Chemotaxis	*Chrysopogon zizanioides* (L.) Roberty-exuded flavonoids	n.d. *	Chemoattraction toward the flavonoids exuded by Pb(II)-stressed plants	[188]
*Rhodococcus* *erythropolis* U23A	Chemotaxis	Concentrated *A. thaliana* root exudates containing flavonoids	n.d. * (flavanone approx. 0.5–1 mM)	Chemoattraction toward *Arabidopsis* root exudates	[189]
*Pseudomonas* *fluorescens* 2P24	Swarming motility Production of cellulose and curli fibers	Apigenin, phloretin	100 µM	Increased swarming motility Up-regulation of flagellar-related genes Up-regulation of cellulose and curli synthetic genes, crucial in EPS production	[37]
*Herbaspirillum* *seropedicae* SmR1	Swimming motility Maize root colonization	Naringenin	100 µM	Promotion of maize root colonization in early phase (first 36 h) Down-regulation of flagellar-related genes and reduced motility possibly due to proximity to the root surface	[190]
*Bacillus subtilis* CIM	Chemotaxis Motility (swimming, swarming and twitching) Growth promotion Biofilm formation	Rutin	1 pM	Chemoattraction Enhancement of swimming, swarming and twitching motility Higher bacterial growth (CFUs) on rutin-supplemented growth medium Induction of biofilm formation	[186]
*Azorhizobium caulinodans* ORS571, *Herbaspirillum* *seropedicae*	Root colonization through lateral root cracks in *Arabidopsis thaliana*	Naringenin, daidzein	50 µM	Promotion of endophytic root colonization performed by nitrogen-fixing bacteria through a Nod factors-independent mechanism	[191]
*Azorhizobium* *caulinodans*, *Azospirillum brasilense*	Root colonization through lateral root cracks in rice	Naringenin	50 µM	Promotion of endophytic root colonization performed by nitrogen-fixing bacteria through a Nod factors-independent mechanism	[192]
*Pseudomonas putida* PML2	Use of flavonoids as carbon sources	Naringenin, quercetin	10 mM	Growth on both flavonoids as unique carbon sources Almost complete depletion of 0.1 mM quercetin added to the medium Elucidation of quercetin degradation pathway in *P. putida*	[182]
*Acinetobacter* *calcoaceticus* MTC 127	Use of flavonoids as carbon sources	(+)-Catechin	3 mM	Growth on catechin as unique carbon source Elucidation of catechin degradation pathway in *A. calcoaceticus*	[193]
*Paraburkholderia* *xenovorans* LB400	Use of flavonoids as carbon sources	Morusin, morusinol, kuwanon C	100 µg/mL	Utilization of the tested flavonoids as sole carbon sources	[194]
Rhizobacteria consortium (*Pseudomonas* sp. + *Stenotrophomonas* sp.)	Use of flavonoids as carbon sources	Flavone, flavanone, isoflavone, 7-hydroxyflavanone, 7-hydroxyflavone, 6-hydroxyflavone	200 µM	Utilization of the tested flavonoids as sole carbon sources	[195]

* Not defined: the specific concentration of flavonoids was not specified because a mixture of root exudates was used for the assay.
